# Visualization of binding patterns for five Leucine-rich repeat proteins in the *Drosophila* embryo

**DOI:** 10.17912/micropub.biology.000199

**Published:** 2019-12-18

**Authors:** Namrata Bali, Kai Zinn

**Affiliations:** 1 Division of Biology and Biological Engineering, California Institute of Technology, 1200 E. California Blvd., Pasadena, CA, 91125, USA.

**Figure 1:**
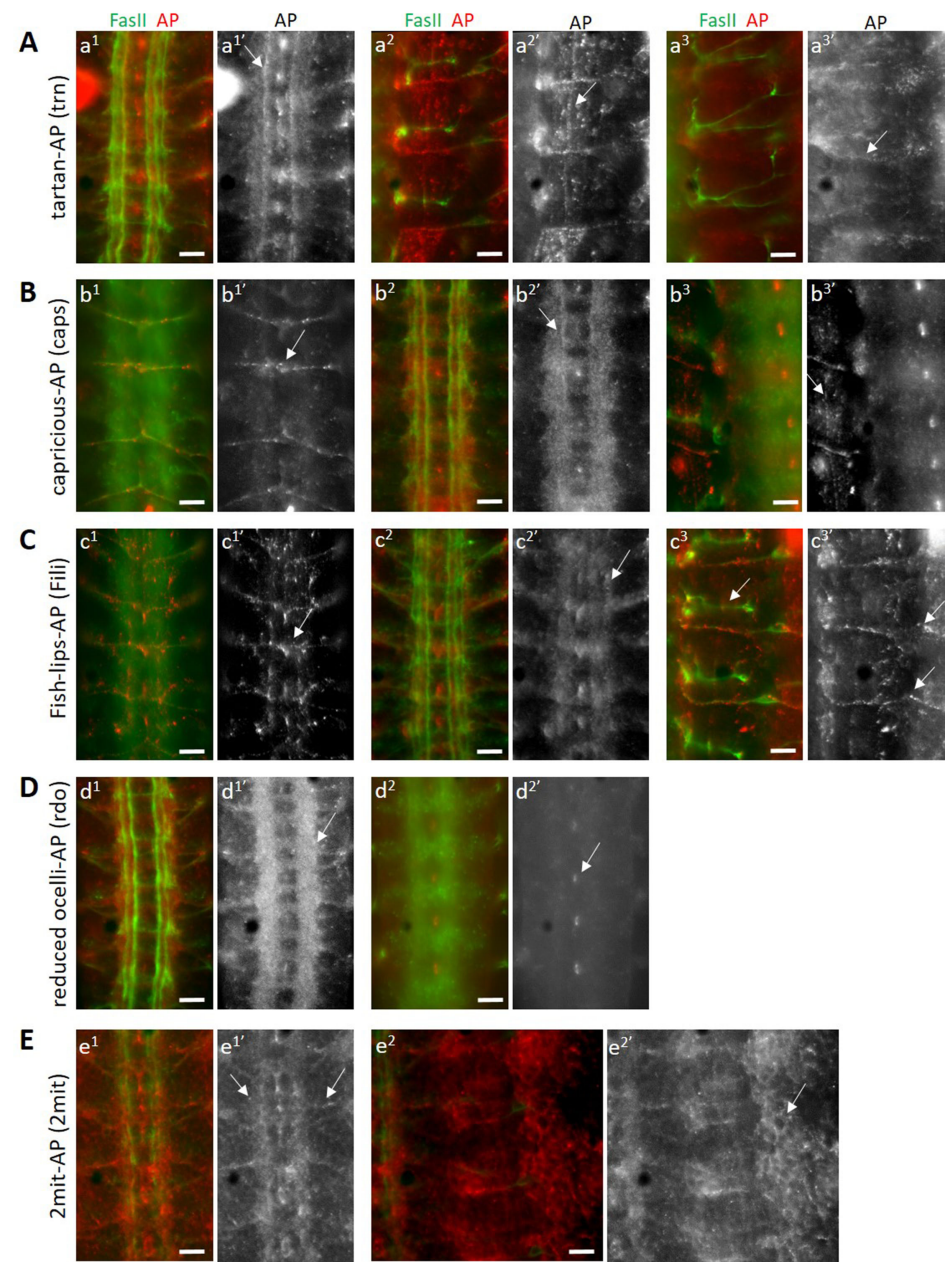
Binding patterns of eLRR AP fusion proteins. All embryos are double-stained for FasII (green) and AP (red). (A) Binding pattern of trn-AP. a^1’ ^shows trn-AP binding to longitudinal axons in the VNC, with stronger binding to one particular axon bundle (arrow). a^2’^ shows trn-AP binding to muscle targets (arrow). a^3’^ shows sensory axons labeled by trn-AP. (B) Binding pattern of caps-AP. b^1’^ shows a set of midline neurons labeled by caps-AP. b^2’^ shows caps-AP binding to longitudinal axons with one axon bundle showing stronger binding (arrow). b^3’^ shows caps-AP binding to muscles (arrow). (C) Binding pattern of Fili-AP. c^1’^ shows Fili-AP binding to dorsal midline neurons (arrow). c^2’^ shows Fili-AP binding to a subset of longitudinal axons. c^3’^ shows Fili-AP binding to the transverse nerve (arrow). (D) Binding pattern of rdo-AP. d^1’^ shows strong rdo-AP binding to longitudinal, commissural and exiting motor axons in the VNC. d^2’^ shows rdo-AP binding to midline glial cells. (E) Binding pattern of 2mit-AP. e^1’^ shows 2mit-AP binding to longitudinal axons and exiting motor axons in the VNC (arrows). Strong binding is also seen to midline cells and fainter staining is seen on the surface of other cells in the VNC. e^2’^ shows 2mit-AP binding to the surface of cells in the periphery (arrow). Scale bars, 10µm.

## Description

Leucine-rich repeat (LRR) domain-containing proteins play central roles in organizing neural connectivity. The LRR is a protein-recognition motif and proteins with extracellular LRR (eLRR) domains mediate intercellular communication and cell adhesion, which in turn regulate neuronal processes such as axon guidance, target selection, synapse formation and stabilization of connections (de Wit *et al.* 2011). The LRR-domain containing Slits and their Robo receptors are one of the best characterized examples of ligand-receptor pairs that regulate midline crossing and axon guidance in both *Drosophila* and vertebrates (Brose *et al.* 1999; Dickson and Gilestro 2006). There are 66 eLRR proteins in Drosophila, many of which are expressed in the nervous system and exhibit strikingly specific expression patterns, often labeling distinct subpopulations of neurons (Lauren *et al.* 2003; Dolan *et al.* 2007). The binding partners and functions of many of these eLRR proteins remain unknown.

We have previously described a novel method to identify ligands and/or binding partners for extracellular proteins (Fox and Zinn 2005; Lee *et al.* 2013; Ozkan *et al.* 2013). This method involves using fusion proteins containing the extracellular domain (ECD) of a protein fused to a pentamerization domain (COMP), followed by human placental alkaline phosphatase (AP). These AP fusion proteins are used to stain live-dissected stage 16 *Drosophila* embryos. The resulting staining patterns can be used as a template to identify expression patterns of the binding partners of the AP fusion protein. Using this technique, we have identified ligands for the receptor tyrosine phosphatases Ptp10D, Lar and Ptp69D (Bali *et al.* 2019; Fox and Zinn 2005; Lee *et al.* 2013). Here, we describe novel binding patterns for 5 eLRR proteins using their respective AP fusion proteins.

Tartan (trn) and Capricious (caps) are two closely-related eLRR proteins with known functions in embryonic motor axon guidance and the innervation of antennal lobe glomeruli by olfactory sensory axons (Kurusu *et al.* 2008; Hong *et al.* 2009). Studies of *trn* and *caps* single and double mutants suggest that the two genetically interact and may function through a common binding partner (Milan *et al.* 2005; Kurusu *et al.* 2008). Tartan may be a substrate for the receptor tyrosine phosphatase Ptp52F (Bugga *et al.* 2009). We stained wild-type live-dissected stage 16 *Drosophila* embryos with trn-AP and caps-AP fusion proteins separately, and found distinct as well as overlapping staining patterns for both fusion proteins. Both trn-AP and caps-AP bind to longitudinal axons in the ventral nerve cord (VNC), with stronger binding seen in one particular axon bundle close to the midline (arrows, a^1’^ and b^2’^). Both also show binding to muscles (arrows, a^2’^ and b^3’^), indicating that they interact with a binding partner expressed on the surface of muscles. trn-AP shows binding to a subset of sensory neurons (arrow, a^3’^), which caps-AP does not. In addition, caps-AP binds to the transverse nerve, which emanates from the midline and is located on the dorsal side of the VNC (arrow, b^1’^).

Fish-lips (Fili) is an eLRR with roles in the regulation of apoptosis (Adachi-Yamada *et al.* 2005) and olfactory receptor neuron (ORN) targeting in the antennal lobe (Xie *et al.* 2019). It is expressed at moderately-high levels during embryonic stages 12 – 17 and during 24 – 48 hours after puparium formation (modENCODE Temporal Expression Data, FlyBase). These developmental stages correspond to peak synaptogenesis times, implying a developmental role of Fili in regulating synaptogenesis. Thus, identification of binding partners of Fili is crucial to understand its roles in CNS development. Staining of wild-type stage 16 embryos with Fili-AP fusion protein shows a restricted binding pattern in the CNS, indicating a similar restricted expression pattern of its binding partners. It binds to a set of dorsal midline neurons (arrow, c^1’^) and a subset of longitudinal axons in the VNC (arrow, c^2’^). A subset of midline cells, putatively glial cells are also labeled with Fili-AP. Strong binding is seen to the transverse nerve in the VNC (c^1^) and in the periphery (arrow, c^3’^), while no labeling is seen to the SNa in the same focal plane (arrow, c^3^).

Reduced ocelli (rdo) is a gene that regulates ocelli development (Caldwell *et al.* 2007) and encodes an eLRR protein of unknown function. Caldwell et al. 2007 showed a broad expression pattern of the encoded protein in the adult nervous system. We performed staining of wild-type stage 16 embryos with rdo-AP fusion protein and found a very strong binding signal in the longitudinal and commissural axons of the VNC (arrow, d^1’^). This binding was limited to the VNC, and no binding was observed to the muscles (data not shown), indicating that the eLRR encoded by rdo interacts with neuronal-specific ligands. We also observed binding in a subset of midline glial cells in the VNC (arrow, d^2’^).

2mit is another gene encoding a putative eLRR and is expressed in the developing nervous system. It has a putative role in regulating short-term memory (Baggio *et al.* 2013). No other information is known about this eLRR. We stained wild-type stage 16 embryos with 2mit-AP fusion protein and saw a wide pattern of binding by this fusion protein, unlike the other restricted patterns observed above. Both longitudinal, commissural as well as exiting motor axons in the VNC are labeled by 2mit-AP (arrows, e^1’^). Moreover, we observed a pan-cellular pattern of labeling in the periphery as well as in the VNC, where 2-mit-AP binding signal is seen on the surface of cells, resulting in a cell-membrane staining pattern (arrow, e^2’^). This implies that the eLRR encoded by 2mit is capable of interacting with ligands expressed on neuronal as well as non-neuronal cell types.

These binding patterns provide clues to the expression patterns of proteins that these eLRRs might interact with to regulate various developmental processes.

## Reagents

y^1^ w^1^ (FlyBase ID FBst0001495)
